# Eliminating Postoperative Nausea and Vomiting in Outpatient Surgery with Multimodal Strategies including Low Doses of Nonsedating, Off-Patent Antiemetics: Is “Zero Tolerance“ Achievable?

**DOI:** 10.1100/tsw.2007.131

**Published:** 2007-06-12

**Authors:** Susan J. Skledar, Brian A. Williams, Manuel C. Vallejo, Patricia L. Dalby, Jonathan H. Waters, Ronald Glick, Michael L. Kentor

**Affiliations:** ^1^ University of Pittsburgh School of Pharmacy and Director - Drug Use and Disease State Management Program, University of Pittsburgh Medical Center, Pittsburgh, PA, USA; ^2^ Department of Anesthesiology, University of Pittsburgh School of Medicine, Pittsburgh, PA, USA; ^3^ Departments of Psychiatry, Physical Medicine and Rehabilitation, and Family Medicine, University of Pittsburgh Medical Center, Pittsburgh, PA, USA

**Keywords:** postoperative nausea and vomiting, PONV, prophylaxis, multimodal prophylaxis, perphenazine, cyclizine, aprepitant, 5-HT3 antagonists, emesis

## Abstract

For ondansetron, dexamethasone, and droperidol (when used for prophylaxis), each is estimated to reduce risk of postoperative nausea and/or vomiting (PONV) by approximately 25%. Current consensus guidelines denote that patients with 0–1 risk factors still have a 10–20% risk of encountering PONV, but do not yet advocate routine prophylaxis for all patients with 10–20% risk. In ambulatory surgery, however, multimodal prophylaxis has gained favor, and our previously published experience with routine prophylaxis has yielded PONV rates below 10%. We now propose a “zero-tolerance” antiemetic algorithm for outpatients that involves routine prophylaxis by first avoiding volatile agents and opioids to the extent possible, using locoregional anesthesia, multimodal analgesia, and low doses of three nonsedating off-patent antiemetics. Routine oral administration (immediately on arrival to the ambulatory surgery suite) of perphenazine 8 mg (antidopaminergic) or cyclizine 50 mg (antihistamine), is followed by dexamethasone 4 mg i.v. after anesthesia induction (dexamethasone is avoided in diabetic patients). At the end of surgery, ondansetron (4 mg i.v., now off-patent) is added. Rescue therapy consists of avoiding unnecessary repeat doses of drugs acting by the same mechanism: haloperidol 2 mg i.v. (antidopaminergic) is prescribed for patients pretreated with cyclizine or promethazine 6.25 mg i.v. (antihistamine) for patients having been pretreated with perphenazine. If available, a consultation for therapeutic acupuncture procedure is ordered. Our approach toward “zero tolerance” of PONV emphasizes liberal identification of and prophylaxis against common risks.

### INTRODUCTION

Despite continued efforts in both treatment and prevention of postoperative nausea and vomiting (PONV), these symptoms continue after both ambulatory and inpatient surgical procedures. Nausea, vomiting, and retching can occur with all types of anesthesia (general, regional, or local). Consequences of PONV can include physical damage, such as rupture of sutures, stitches, and esophageal tissue, and metabolic problems, such as electrolyte imbalances and dehydration[1,2]. Patients experiencing PONV are dissatisfied with care, and may experience loss of work and delayed hospital discharge, all of which can lead to increased direct and indirect health care costs. In severe cases of PONV, although rare, aspiration of gastric contents may occur, resulting in pulmonary sequelae, such as pneumonia or pneumothorax[3,4]. The incidence of PONV 24 h after a surgical procedure (also called postdischarge nausea and vomiting or PDNV) continues to be in the range of 25–30%[5], with numbers reported as high as 70% in higher-risk patients[6].

It is important not to overlook the hidden costs of the rescue treatment of such a common complication. Williams et al. reported that same-day discharge times after ambulatory knee surgery were delayed by 27–45 min whenever a patient encountered at least one symptom requiring a nursing intervention[7]. Whether the anticipated PONV rate is low or high, it is well known that any PONV or PDNV has the potential to delay processes of patient care. It has been reported that patients who were sufficiently symptom free on operating room exit (no immediate PONV or pain, and rapid awakening) were able to bypass the traditional recovery room and achieve successful same-day discharge with high frequency[8]. In these patients, recovery room bypass was associated with a $400 per patient hospital cost savings, while unplanned hospital admission was associated with a $400 hospital cost increase[8].

The objective in this manuscript is to supplement the conclusions of recent evidence-based PONV guidelines[2,9,10,11] with clinical practice experience and study from an ambulatory surgery center at our facility. Incorporating "quality improvement" concepts in rational care decisions has been endorsed by Berwick[12]: "Our work in medicine should be grounded in sound evidence, and proper designs and statistics are basic to this quest. But, buried in our embrace of classical rules of design lies a problem of significant and growing scale; namely, not all sound learning occurs through formal science. Our rules about what shall and shall not be cited and published as science may have become too stringent for our own good."

### PREVENTION OF PONV

#### Avoidance of Volatile Anesthetics in Outpatients

We concur with earlier work denoting that the most effective intervention in preventing PONV in outpatients is the avoidance of volatile anesthetics. The evidence basis for this statement is well substantiated, but may not be particularly well publicized, since volatile agent use remains ubiquitous in outpatient surgery. Apfel et al. declared that volatile agents are the main cause of early PONV[13]. Sneyd et al. reported in a meta-analysis that maintenance of anesthesia with volatile agents has a significantly higher incidence of PONV when compared with anesthetic maintenance with propofol (odds ratio 2.7)[14]. Given this recommendation, the remainder of this manuscript will provide a perspective into multimodal PONV prevention, and will present a strategy for low-cost routine prophylaxis followed by higher-cost rescue, in an effort toward elimination of PONV in surgical outpatients.

#### Avoidance of Opioids

It is well known that opioids induce PONV. The equivalent of a 3–4 mg morphine increment has been associated with one additional, clinically meaningful, opioid-related symptom, or one additional patient day with an opioid-related, clinically meaningful event[15]. Relevant options to reduce opioid use during surgery and opioid requirements after surgery include regional anesthesia techniques[16,17,18]. PONV is less likely in certain situations when clonidine is used as an analgesic adjunct[19,20] and the same is also noted in a recent gabapentin meta-analysis[21].

Other risk-reduction strategies proposed include avoiding nitrous oxide, minimizing intraoperative neostigmine (especially large doses greater than 2.5 mg i.v.), preferential use of total intravenous and/or regional anesthesia, and adequate patient hydration[11]. Sugammadex[22], the new neuromuscular blockade reversal agent, appears to be "relatively" antiemetic, since it can be used instead of neostigmine. It is also important to note that neither Sneyd et al.[14] nor Apfel et al.[9] have found that nitrous oxide increases PONV risk when coadministered with propofol as the maintenance anesthetic. Nonpharmacologic interventions have become the standard of care for all patients, but by themselves do not reduce the rate of PONV to a significant extent.

### A Call for Routine Prophylaxis with Three Nonsedating, Low-Cost Agents

Current evidence-based strategies for prevention of PONV include: (1) proactive risk assessment, (2) avoiding PONV "triggers", and (3) administration of prophylactic antiemetic medications. In his review, Gan summarized risk factors for PONV in adults and children, and concluded that several key independent risk factors are well established as predictive for PONV[2] (Fig. 1). This report also describes other possible risk factors, such as intense preoperative anxiety, certain surgery types (e.g., intra-abdominal, orthopedic, major gynecologic), increased duration of anesthesia, and general vs. regional anesthesia[2]. Risk factors are reported as being related to the patient, the surgical procedure, and the anesthetic regimen, a common method of determining risk.

***Independent, "Well-Established" Risk Factors that Can Be Modified by Anesthesiologist***

- Use of volatile anesthetics
- Intraoperative or post-operative opioids
- Use of large-dose neostigmine (greater than 2.5 mg IV)
- Use of nitrous oxide (with volatile agents)

***Independent, "Well-Established" Risk Factors than Cannot Be Modified by Anesthesiologist***

- Increased duration of surgery
- Female gender (i.e., after puberty)
- Non-smoking status
- History of motion sickness or PONV
- Childhood/young adulthood

**FIGURE 1.** Risk Factors for PONV (adapted from Gan, 2006[2])

Defining which factors are most associated with emetic risk can be a major challenge to practitioners today. Risk assessment can set the basis for evidence-based recommendations for managing PONV, but the parameters for triggering routine prophylaxis with available off-patent antiemetics have not been well studied. Apfel et al. identified a simplified risk assessment scoring system to determine PONV risk to include four patient-related risk factors: female gender, nonsmoking status, history of PONV or motion sickness, and postoperative use of opioids[23]. Koivuranta et al., using a survey design, created a five-factor risk assessment including female gender, history of PONV, duration of surgery greater than 60 min, nonsmoking status, and history of motion sickness[24]. The incidence of PONV increases with each risk factor a patient possesses. A pediatric scoring system has been designed by Eberhart et al.[25]. Gan reports that these scoring systems, including the other six scoring systems available in the literature, achieve approximately a 12–57% improvement over guesswork in prevention of PONV, and recommends the Apfel, Koivuranta, and Eberhart systems for their simplicity and accuracy[2,23,24,25]. Importantly, Apfel and Koivuranta determined PONV risk rates in the settings of all patients receiving volatile agents and airway devices[23,24]. These two risk calculation methods do not account for the independent risk factors of volatile agents and airway devices, given that alternatives are available (such as propofol for maintenance of sedation or anesthesia, and/or use of regional anesthesia without general anesthesia/airway device).

Smaller studies have shown that proactive risk assessment with prophylactic antiemetics still yields PONV rates approximating 20%. As a result, practitioners of ambulatory anesthesia should continue to search for methods to eliminate PONV. Korhonen et al. evaluated 62 patients undergoing outpatient knee arthroscopy with desflurane vs. small-dose bupivacaine spinal[26]. In this study, desflurane patients were pretreated with ondansetron if two or more risk factors (risk scores of 40% or higher) were reported (the planned use of desflurane was not included as a risk factor)[26]. Despite prophylaxis for "at-risk" patients, 19% of desflurane patients encountered PONV (95% confidence interval: 5%, 32%), vs. none in the bupivacaine spinal group.

It is our opinion that many practitioners of anesthesia tend toward nonaggressive prophylaxis when determining PONV risk preoperatively. Even if patients have a baseline 10–20% risk for PONV based on risk scoring (i.e., 0–1 risk factors), we consider this risk as unacceptably high, given the desire of patients to avoid this symptom[27] and the availability of nonsedating, off-patent antiemetics. These authors consider this an ethical dilemma with respect to the withholding of effective interventions for a high-probability occurrence.

### Authors' Institutional Experience

Based on reports from the late 1990s indicating that patients want to avoid PONV[27], we now review our experience in outpatient orthopedic surgery in which regional anesthesia is preferentially used. Kentor and Williams reported the same-day outcomes on 1907 orthopedic patients (from 1995 to 1999) in a quality improvement–based clinical pathway[28]. These authors commonly used a "pan-prophylaxis" technique (before risk scoring strategies were widely published and/or endorsed) and retrospectively compared these "pan-prophylaxed" patients with patients given less-systematic prophylaxis by prescriber preference at our institution. These inquiries about pan-prophylaxis verified the reports of Sneyd et al.[14] and Apfel et al.[13] that patients receiving volatile anesthetic agents are at additional risk (25–33% incidence of PONV), as are patients receiving regional anesthesia without antiemetic prophylaxis (20% incidence with 0–1 prophylactic antiemetic)[28]. When volatile agents were avoided, and when regional anesthesia techniques were combined with two or more antiemetics (perphenazine 1.25 mg i.v. and dexamethasone 4 mg i.v. as the most common strategy used in this review period), the PONV incidence was only 9% (95% confidence interval: 7–11%)[28]. Based on this work, we now consider less than 10% as the "acceptable threshold" for PONV incidence and aim for less than 5% in daily practice.

After reviewing these trends favoring "pan-prophylaxis" without regard for patient-based risk factors, the antiemetic combination of perphenazine-dexamethasone-ondansetron was applied to a prospective cohort of patients undergoing spinal/regional anesthesia for anterior cruciate ligament reconstruction (ACLR) at our institution[29]. In this study, the PONV rate was a low 4% (95% confidence interval: 2–7%), representing a significant improvement over previous work in our institution in which regional anesthesia patients for ACLR encountered an unacceptable PONV rate of 17%[30]. For reference, our institution's ACLR patients (1996–1999) who received general anesthesia with volatile agents, no antiemetics, and no nerve blocks, had a PONV rate of 33%, and an unplanned hospital admission rate of 17%[8].

We acknowledge that our institutional rates of PONV (via audit) may be lower than rates reported in the literature. This may be for one of several reasons. First, our management-oriented definition of PONV (in our previous reports) was the presence of a documented nursing intervention on the patient's medical record; this is in contrast to a prospectively collected patient report of PONV not necessarily linked to an intervention in the medical records. In addition, many prospective PONV studies enroll patients who are at a high baseline risk, whereas our audits were of all patients regardless of baseline risk. These reasons may sufficiently describe discrepancies in PONV rates in our reported audits vs. in randomized controlled trials, however, our goal with this review is to reinforce, in our opinion, that the concept of "low risk for PONV" being 10–20% is still too high.

### Evidence Basis for PONV Prophylaxis Toward "Zero Tolerance"

There currently is no "gold standard" for prophylaxis against PONV. Multimodal prophylaxis involving both pharmacologic and nonpharmacologic interventions, as well as risk reduction, has been recommended for PONV prophylaxis[6].

In this manuscript, we recommend that the ideal prophylactic antiemetic plan for outpatients should consist of: (1) the avoidance of volatile anesthetics, using propofol as the "induction" and "maintenance" anesthetic of choice; (2) avoidance of opioids and maximizing the use of nonemetogenic multimodal analgesics; (3) the use of local and regional anesthesia, and multimodal analgesia, to the fullest extent possible; and (4) the routine, low-dose administration of selected, off-patent antiemetics (to be listed later in the text) in patients for whom there are no contraindications. Evidence for prophylaxis toward "zero tolerance" is presented in the following paragraphs.

The theory with use of multiple antiemetic agents is to block more than one of the major receptor systems that are part of the emetic pathways involved in PONV[1]. Benefit has been shown with multimodal (>2) and dual strategies for PONV prevention. Habib et al. and Scuderi et al. have studied multimodal management in randomized trials in laparoscopic surgery and found that this strategy was associated with higher response rates than conventional regimens[10,31]. In general, they found that combination therapy is more effective than monotherapy and using agents that affect different emetic receptors is beneficial[10,11,31]. Habib et al. found that the best outcomes were achieved with the combined use of propofol, droperidol, and ondansetron (90% complete response at 2 h and 80% complete response at 24 h)[10]. Scuderi et al. reported that the best outcomes (1/60 with nausea, and no vomiting, 95% confidence interval: 0–5%) were achieved with a multimodal strategy consisting of droperidol, dexamethasone, ondansetron, propofol, remifentanil, ketorolac, low-dose fentanyl for transitional analgesia, and anxiolysis and hydration[31]. With this algorithm, patients achieved a 98% complete response on the day of surgery, and an 88% complete response rate at 24 h. Eberhart et al. reported a PONV incidence of 7% (95% confidence interval: 3%, 14%) in high-risk women undergoing gynecologic or breast surgery when combination therapy consisted of propofol, dexamethasone, haloperidol, and tropisetron[32]. Interestingly, one study by Hammas et al. that used four-drug multimodal prophylaxis (propofol induction and infusion, with dexamethasone, droperidol, and metoclopramide), but also used intraoperative sevoflurane, reported PONV rates of 10–14% after breast surgery and 20–30% after abdominal surgery, when assessed at four different intervals during the first 24 postoperative hours[33]. It seems likely that if this protocol had avoided sevoflurane and added a 5-$HT_3$ receptor antagonist, the outcomes would have possibly approached the reports of the other authors above[10,31,32].

A large, multicenter factorial study by Apfel et al. of interventions to prevent PONV showed that droperidol, dexamethasone, and ondansetron relatively reduced PONV (from the previous risk calculation) to same effect, and addition of a second agent resulted in an absolute benefit that was less that the initial intervention[9]. These authors summarized this concept by stating that each additional antiemetic prophylaxis intervention (of a unique central mechanism) reduces PONV risk by 22–26%. For patients who did not receive any prophylactic agent, the PONV rate was 52%. Therefore, patients with 10% baseline risk receiving one, two, or three prophylactic antiemetics can expect PONV rates of 7.4, 5.5, and 4%, respectively. For patients at highest risk, a multimodal strategy has been advocated[9,11,33,34]. However, it is our opinion that 10% risk (in patients with “no risk factors" as defined by Apfel et al.[23]) is sufficiently “high risk”, warranting routine prophylaxis with low-cost, low-dose, nonsedating medications (such as ondansetron, dexamethasone, and either perphenazine or cyclizine) in order to maximize patient satisfaction and minimize costly delays in recovery.

### MECHANISTIC CONSIDERATIONS OF PROPHYLACTIC ANTIEMETICS

Combinations of pharmacological agents are useful, as there are several receptor systems involved in the development of PONV. No currently available agent will antagonize all receptor sites involved in the antiemetic response. Currently available classes of medications available to treat PONV include phenothiazines, antihistamines, butryophenones, anticholinergics, benzamides, dexamethasone, and serotonin (5-$HT_3$) antagonists. Newly available classes include next-generation 5-$HT_3$ antagonists and neurokinin antagonists. The cheapest, safest, most effective, longest-duration antiemetics should be the agents of choice in PONV prophylaxis. Our recommendations for dosing of antiemetic agents for use in “zero-tolerance” prophylaxis and treatment for outpatients are provided in Table 1.

Our recommendation is to use PONV prophylaxis (with low-cost agents) more aggressively than currently stated in existing consensus statements, and to continue to build evidence about newer and/or higher-cost agents, and associated therapeutic indices, for rescue (Fig. 2).

#### Phenothiazines

Phenothiazines (PTZ) block dopaminergic receptors in the chemoreceptor trigger zone (CTZ). Prochlorperazine, promethazine, and perphenazine are commonly used PTZ. A disadvantage of prochlorperazine, chlorpromazine, and promethazine is that they can produce significant sedation, lethargy, and hypotension, and they may also require repeated dosing. Of note, perphenazine is the only PTZ of those listed that is not associated with sedation or hypotension at usual antiemetic doses[35]. As a result, perphenazine (oral form) remains a routinely used prophylactic antiemetic in our institution.

Extrapyramidal symptoms (EPS) from PTZ, ranging from restlessness to oculogyric crisis, may occur[36]. With this side effect profile, particularly the excessive sedation, intravenous use of prochlorperazine, chlorpromazine, and promethazine is limited in the ambulatory setting[11]. The use of low-dose promethazine (5–10 mg i.v.) has gained some popularity recently both for prophylaxis and for rescue, due to its additional antihistamine effects[37,38,39]. If PONV prophylaxis previously failed with perphenazine, then cyclizine (antihistamine) may be a logical premedicant instead, since cyclizine increases lower esophageal sphincter tone[40]. Perphenazine and cyclizine are only available for oral administration in the U.S., which may make their use before surgery more valuable when the intravenous form is not available. Unlike cyclizine, traditional antiemetics droperidol and promethazine reduce lower esophageal sphincter tone[40].

Drug product shortages and discontinuations have affected availability of these older antiemetic agents. When a shortage of prochlorperazine was encountered, Sheth et al. reported adverse events with promethazine (which was used as a hospital-approved therapeutic substitution)[41]. Promethazine has both antihistaminic and anticholinergic effects, and produces significant sedation in patients on concomitant opioids, providing further evidence limiting the utility of this agent as a routine outpatient antiemetic[41].

**TABLE 1**
**Dosing Information for Multimodal Antiemetic Prophylaxis and Rescue in Adult Surgical Outpatients* (Based on the Agents Described in Fig. 2; Applicable to Nonparturients, Adults Less than 70 Years Old**, and Not Undergoing Cataract Surgery***)**

| | Therapeutic Class and Effects | Dosage** | Administration Time | Comments |
|---|---|---|---|---|
| **Antiemetic Agent** | | | | |
| Dexamethasone (step 1 agent of choice) | Class: steroid<br>Effects: antagonize prostaglandin, endorphin release, appetite stimulation | 4 mg i.v. | At induction of anesthesia or after ample sedation | -Can cause perineal pruritis if not preceded by anesthetic induction or ample sedation<br>-Flushing, hyperglycemia, adrenal suppression<br>-Contraindicated in diabetics, but insufficient data to validate<br>-Documented value in reducing PDNV (in combination with 5-$HT_3$ antagonists) |
| Ondansetron (step 1 agent of choice) | Class/Effect: 5-$HT_3$ receptor antagonist | 4 mg i.v. | Immediately before end of surgery | -Headache, increased liver enzymes<br>-Ineffective with repeated doses<br>-Documented value in reducing PDNV<br>-Off-patent and inexpensive |
| Perphenazine (step 1 option) | Class: PTZ<br>Effects: antidopaminergic, antihistaminic, anticholinergic | 8 mg oral | Immediately on arrival to the same-day surgery unit (before surgery begins) | -Substrate and inhibitor of CYP 2D6<br>-Avoid in patients with Parkinson's disease, history of EPS with other antipsychotics, or if taking antidysrhythmics or other cardiac medications that are metabolized by CYP 2D6 (e.g., propafenone)<br>-Rescue with haloperidol 1 mg i.v. can be considered, but may increase risk of sedation and/or EPS |
| Cyclizine (step 1 option) | Class: antihistamine<br>Effects: antihistaminic, anticholinergic, antidopaminergic | 50 mg oral | Immediately on arrival to the same-day surgery unit (before surgery begins) | -50-mg i.v. dose efficacious as monotherapy compared with placebo; intravenous formulation not available in U.S.<br>-Intravenous formulation precipitates when mixed with ketorolac<br>-May induce EPS rarely at the 50-mg i.v. dose<br>-Increases lower esophageal sphincter tome |
| Scopolamine (step 1 option) | Class: anticholinergic<br>Effect: anticholinergic | 1.5 mg transdermal patch | Immediately on arrival to the same-day surgery unit (before surgery begins) or rescue | -Do not cut the patch<br>-Long onset of effect<br>-Anticholinergic effects in the elderly<br>-Contraindicated in narrow-angle glaucoma<br>-Central nervous system effects in the elderly and in those with renal or hepatic dysfunction |
| Aprepitant (step 2) | NK-1 antagonist | 40 mg oral | Within 3 h of induction of anesthesia | -Expensive for prophylaxis; potential additive/synergistic role with other low-cost antiemetics warrants additional study |
| **Rescue Antiemetic Techniques, Agents, and Sequence** | | | | |
| Acupuncture techniques | | See text | | -If available promptly after consultation of subspecialty service, would be the rescue technique of first choice after failure of step 1 pharmacologic antiemetic techniques above<br>-Logically attempted before considering unplanned hospital admission for refractory PONV |

### TABLE 1 (continued)

| | | | | |
|---|---|---|---|---|
| Haloperidol | Class: butyrophenone<br>Effect: antidopaminergic | 1–2 mg i.v. | Rescue (especially if perphenazine was not used preoperatively) | -Duration of effectiveness shorter than that for droperidol<br>-Sedation, "lock-in"<br>-FDA Black Box warning with droperidol related to $QT_c$ prolongation and torsades de pointes[27]<br>-Due to short duration, neither haloperidol nor droperidol appear to reduce PDNV<br>-Droperidol not effective in men, haloperidol not similarly studied |
| Alternative 5-$HT_3$ antagonist<br>Dolasetron (D)<br>Granisetron (G) | Class/Effect: 5-$HT_3$ receptor antagonist | 12.5 mg i.v. (D); 0.1–1 mg i.v. (G) | Rescue | -Headache (D,G) and increased liver enzymes (D)<br>-Ineffective with repeated doses, most logical rescue agent may be G when ondansetron has failed, based on cytochrome P450 metabolic characteristics<br>-Documented value in reducing PDNV |
| Promethazine | Class: antihistamine<br>Effects: antihistaminic, anticholinergic, antidopaminergic | 6.25 mg i.v. | Rescue (especially if cyclizine was not used preoperatively) | -Verify patency of intravenous cannula due to risk of vesicant-induced tissue necrosis<br>-Doses above 6.25 mg likely to worsen sedation-related side effects and opioid-related drug interactions (somnolence)<br>-Reduces lower esophageal sphincter tone |

* In addition to propofol induction and maintenance, avoidance of opioids, use of locoregional and multimodal analgesia, and complete avoidance of volatile agents.

** Dose reductions by 50–100% may be recommended for patients 70 years and older, depending on the extent of coexisting disease.

*** No prophylactic antiemetics are recommended for cataract surgery performed under monitored anesthesia care unless PONV was previously encountered while undergoing a similar procedure.

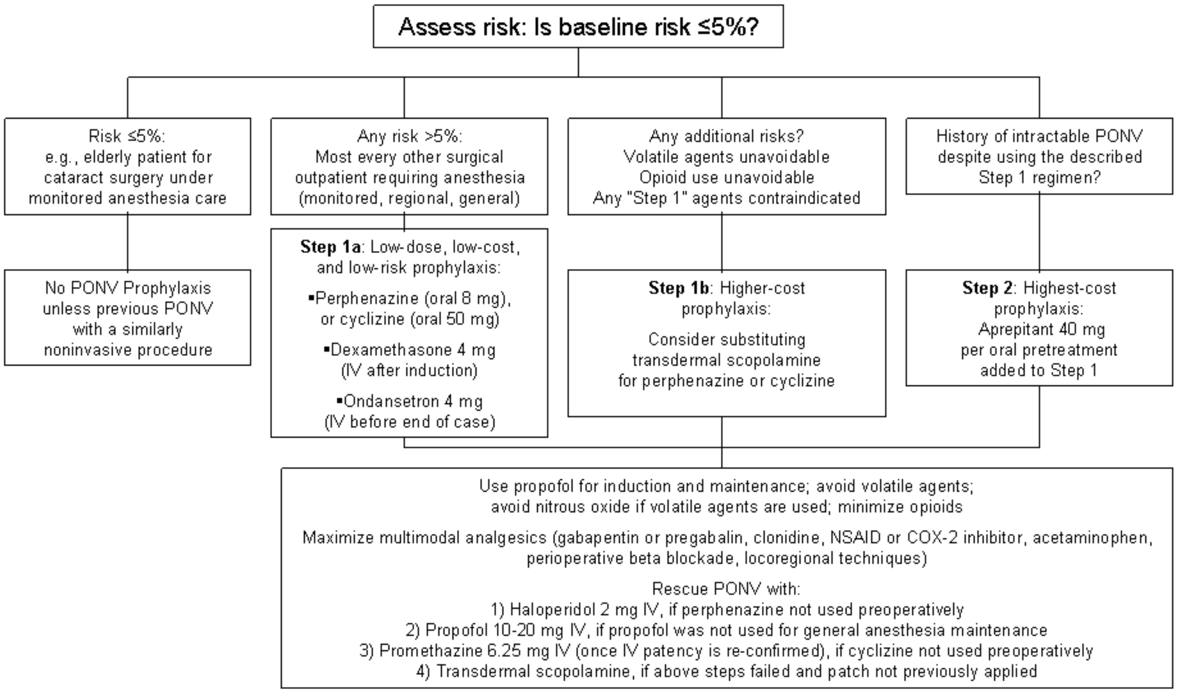

**FIGURE 2.** PONV in outpatients (prophylaxis and treatment): a "zero-tolerance" strategy proposal.

Perphenazine, although no longer available in intravenous form, has been shown to be an effective antiemetic in 5-mg i.v. doses given before induction of anesthesia[35]. Perphenazine (1.25–2.5 mg i.v.), combined with dexamethasone (4–10 mg i.v.) has also been shown to be effective for PONV prophylaxis in orthopedic surgical outpatients[28,30]. In the authors' clinical experience, this combination was associated with protection against PDNV as well[28], but further study is needed to verify. Recent studies have shown effectiveness of an equivalent 8-mg oral regimen of perphenazine, given before surgery (with dexamethasone and ondansetron given during surgery)[29]. Perphenazine is a substrate for the cytochrome P450 (CYP) 2D6 isoform and also inhibits 2D6. Premedication with perphenazine may, in theory, enhance the efficacy of some 5-$HT_3$ antagonists, such as ondansetron and dolasetron (both of which are also substrates of CYP 2D6). Further study is needed to document if this is true.

It is important to note that perphenazine is not indicated in patients with previous adverse reactions to other PTZ. Perphenazine is contraindicated in patients with Parkinson's disease, as are other antidopaminergic agents. Although the oral dosing for the antiemetic indication in adults is recommended as 8–16 mg/24-h period[42], in a study by Williams et al.[29], two patients (both 105 kg) who received 16 mg of oral perphenazine (in divided doses), encountered EPS in the first 2 days after surgery. Thus, our recommendation is for a routine preoperative perphenazine oral dose of 4–8 mg (to which we have never had an adverse reaction in our institution, to our knowledge). When perphenazine was available in intravenous form, in our usage experience, we had no observed EPS in over 1700 clinical patients given a single dose of 1.25–2.5 mg i.v. for PONV prophylaxis.

It is well described that pharmacologic PONV rescue should be attempted with an antiemetic agent from a different pharmacologic class than the prophylactic drug[11,43,44]. Pharmacologically, when using a "full-dose" PTZ or antihistamine (e.g., perphenazine or cyclizine orally before surgery, see Table 1), it may not be prudent to rescue PONV with another full-dose antidopaminergic and/or antihistaminic agent (e.g., promethazine). Consideration for rescue with a single, low-dose antidopaminergic (haloperidol 1–2 mg i.v.) appears reasonable (Table 1). If rescue with haloperidol fails, however, after prophylaxis with an antidopaminergic (perphenazine), an antihistamine (cyclizine), and a 5-$HT_3$ antagonist, attention is logically directed to other PONV rescue techniques, such as acupuncture (to be described later).

### Butyrophenones

Butyrophenones are dopamine receptor antagonists, similar to PTZ. Droperidol and haloperidol are two commonly used butyrophenones. In the treatment of PONV, more experience is with droperidol. Dosing for droperidol for PONV is 0.625–1.25 mg i.v. (not exceeding 2.5 mg) in adults and 50–75 mcg/kg in children[11,45]. At doses of 1.25 mg i.v., the incidence of side effects (such as sedation, dizziness, hypotension, dysphoria, and EPS) does not differ significantly compared with ondansetron[11,46]. Prior to 2001, when the Food and Drug Administration (FDA) box warning was added to droperidol product labeling to denote risk for torsades de pointes and $QT_c$ prolongation[47], consensus guidelines and pharmacoeconomic analyses recommended droperidol as a first-line antiemetic for PONV management[45,48]. In their 2003 PONV consensus guidelines, Gan et al. noted that if the box warning were not placed on droperidol in 2001, it would have been the panel's first-line recommendation for PONV prophylaxis[11]. A recent review by McKeage et al. reiterates droperidol's similar efficacy to ondansetron and dexamethasone, and notes that its adverse event incidence profile is similar to ondansetron and that of placebo[49]. In light of the warnings and controversy surrounding droepridol's use, however, creation of treatment algorithms recommending its use have become problematic. Interestingly, in a statistical analysis involving the interaction of factors in a regression analysis, Apfel et al., in 2004, showed that droperidol did not significantly reduce the PONV risk in men, when compared with women[9].

Haloperidol has been recently reviewed[50], with these authors concluding (based on a meta-analysis of studies having enrolled 1000 adult controls and 1400 treated adults having received haloperidol) that low-dose haloperidol (1–2 mg) is an effective antiemetic, but with a far shorter duration than with droperidol. This shorter duration of haloperidol seems likely to render it as not useful for the prevention of PDNV. Extrapyramidal reactions occurred in one patient with a 4-mg i.v. dose and sedation occurred in 25% of patients with the 5-mg i.v. dose. Haloperidol, 1 mg i.v., given early in surgery may be an equally effective antidopaminergic option to 8 mg perphenazine orally given before surgery, but is likely to have a shorter duration. In either case, further research is needed to compare the two agents. A 1-mg i.v. dose of haloperidol appears to be safely repeated postoperatively, if rescue is required (Table 1), but postoperative haloperidol dosing has not been studied in patients receiving perphenazine pretreatment.

Caution is advised in patients taking certain psychiatric medications since haloperidol, perphenazine, and cyclizine are CYP 2D6 inhibitors and substrates. Further study is needed to determine comparative efficacy of these described doses, along with (likely rare) associated side effects. In high-risk populations, haloperidol 1 mg i.v. as monotherapy appears to have only short-term benefit (0–2 h), with no meaningful benefit after that[51].

### Antihistamines

Antihistamines act on the vomiting center and vestibular pathways. Examples are dimenhydrinate (which consists of diphenhydramine and chlorotheophylline), hydroxyzine, cyclizine, and diphenhydramine. Promethazine (discussed earlier) also has significant antihistaminic properties. Antihistamines are useful in prophylaxis and treatment of motion sickness, but some can cause significant sedation. Anticholinergic effects, such as dry mouth, visual disturbances, and sedation, can make these agents dangerous for the elderly population.

Cyclizine, a piperazine antihistamine, has emerged as a potentially clinically relevant prophylactic antiemetic based on previous European experiences even before the withdrawal of droperidol. Cyclizine has shown (at 50 mg i.v.) greater efficacy than perphenazine (2.5 mg i.v.)[52,53], equal efficacy to perphenazine (5 mg i.v.)[52], more efficacy than dexamethasone (8 mg i.v. in parturients having received intrathecal morphine)[54], and equal efficacy to ondansetron[55] and droperidol[56], when comparators were administered as monotherapy. Finally, cyclizine increases sphincter tone[40], reduces gastric dysrhythmias, and is less sedating than is dimenhydrinate[57]. Cyclizine, which has antidopaminergic and anticholinergic effects, has been shown in recent case reports to be associated with dystonic reactions in the 50-mg i.v. dose. After originally postulating that patients with central nervous system disease may be at higher risk for these dystonic reactions[58,59,60], there have also been reports of otherwise healthy patients also experiencing dystonic reactions associated with cyclizine use (at 50 mg i.v.)[61,62]. The antidopaminergic effects and potential for dystonic reactions with cyclizine (based on the reports above) may make its use ill-advised in patients with Parkinson’s disease or history of EPS.

As of yet, no dose-finding studies for cyclizine have been performed and injectable cyclizine is no longer available in the U.S. Since dystonic reactions have been seen at the 50-mg i.v. dose, we recommend dosing of cyclizine as 50 mg orally before surgery (see Table 1). The anticholinergic effects of cyclizine would make its use a poor choice in patients in whom acute, significant, cardiac disease is present, when tachycardia is to be avoided at all costs. Previously, when the intravenous formulation was available, it was important to know that injectable cyclizine and ketorolac form a precipitate when mixed together[63]. Selection of perphenazine over cyclizine primarily depends on patient past medical history and present comorbidities, and possibly is related to previous PONV despite perphenazine pretreatment. Our limited experience in patients receiving both perphenazine and cyclizine as simultaneous pretreatment indicates that significant (unwanted) sedation can occur (personal communication, author BAW).

### *Benzamides*

Benzamides have central and peripheral antiemetic properties. Metoclopramide is an example of a benzamide. Benzamides block dopaminergic receptors at the CTZ, and they also lower esophageal sphincter tone (opposite of cyclizine). Benzamides enhance gastric and small bowel motility, and prevent delayed gastric emptying of opioids. Results of metoclopramide studies are variable due to differing routes of administration, time between administration and induction and recovery from anesthesia, type of procedure, and anesthetic drugs and techniques used in the operation. In a systematic review of randomized, placebo-controlled studies of metoclopramide conducted by Henzi and colleagues, it was found that metoclopramide cannot be recommended for PONV prevention due to its being less effective than other available agents and notable adverse event profile that may include sedation, weakness, and EPS[64]. In additional recent reviews of the literature, metoclopramide cannot be recommended for PONV prevention or management[1,11].

### Anticholinergics

Anticholinergic agents, such as scopolamine, exert their antiemetic effect by antagonizing muscarinic and histaminic receptors in the vestibular and vomiting centers. Adding atropine or scopolamine to an opioid will decrease emesis[48]. The transdermal scopolamine patch is FDA approved for PONV prevention as a 1.5-mg, 72-h patch, applied the evening prior to surgery or 4 h before the end of anesthesia or surgery[65]. It has been shown to be helpful in controlling the incidence of severe nausea and vomiting, especially in patients with a history of motion sickness. Side effects with scopolamine, such as visual disturbances, dysphoria, confusion, and restlessness, can occur, especially in the elderly or those with compromised end-organ function. The transdermal product takes approximately 2–4 h to exert its effect, which limits its utility for nonelective surgeries. Additionally, transdermal scopolamine is contraindicated in children and has not been studied sufficiently in elderly patients to recommend its use for PONV in those populations[65,66]. A systematic review by Kranke et al. studied the safety and efficacy of transdermal scopolamine for PONV prevention and concluded that it was an effective antiemetic agent[66]. Attention to its dosing, application of the patch, and contraindications must be paid before use of this agent. As noted earlier, it should be noted that both perphenazine and cyclizine have anticholinergic properties. As a result of the anticholinergic effects of other recommended prophylactic antiemetics, along with the side effect profile of scopolamine, we do not believe that the routine use of a scopolamine patch has a place in PONV prophylaxis when the less-sedating perphenazine and/or cyclizine are used. That said, the scopolamine patch may be a logical routinely administered antiemetic when perphenazine and cyclizine are not used, especially due to the sustained PDNV prophylaxis provided by the scopolamine patch. Studies comparing the side effect profile of perphenazine/cyclizine vs. the scopolamine patch have not been performed.

The scopolamine patch is likely a very logical first agent when an unplanned hospital admission has occurred for the treatment of refractory PONV, once the anticholinergic crossover effects of perphenazine or cyclizine have passed, or possibly for sustained postoperative antiemesis after discharge home for patients deemed at high risk for PDNV in situations where oral ingestion of perphenazine (e.g., 4 mg every 12 h) or cyclizine (50 mg daily) are not practical. These authors maintain that the principle of multimodal antiemesis (addressing the most possible central mechanisms) logically favors prophylaxis with the low-cost, nonsedating perphenazine or cyclizine (each of which covering two or three of the antidopaminergic, antihistaminic, and anticholinergic mechanisms) vs. higher-cost and more-sedating scopolamine (which only addresses the anticholinergic mechanism). Certainly, confirmation studies are required.

### Dexamethasone

Dexamethasone has been used historically for both prevention and treatment of chemotherapy-induced nausea and vomiting, so its study for PONV management is intuitive, especially in the face of product discontinuations and shortages with traditional antiemetic agents. Use of dexamethasone as a single dose, administered before or at anesthesia induction (vs. at the end of the procedure) has been shown effective for PONV reduction, with a number needed to treat (NNT) of 4.3 for prevention of late PONV and 7.1 for PONV in adults[11,67,68]. A meta-analysis by Eberhart et al. showed that the combination of dexamethasone with another antiemetic was superior to dexamethasone alone for PONV[69]. Meta-analyses have shown that combination of dexamethasone/5-$HT_3$ (and also combination of 5-$HT_3$/droperidol) was more effective at PONV prevention than single-agent prophylaxis with a 5-$HT_3$ agent[70] and that prophylaxis with 5-$HT_3$/dexamethasone compared to dexamethasone alone resulted in lower use of rescue antiemetics ($OR_{pooled}$ = 0.48)[71]. An evaluation of dexamethasone i.v. and ondansetron i.v., alone or in combination in outpatient surgery, showed that postdischarge vomiting was significantly reduced when the drugs were used in combination[72]. The optimal dose of dexamethasone for PONV prevention is still under study, in the range of 2.5–10 mg. The 8-mg dose appears to be the most common adult dose studied currently[67], although we suggest using a 4-mg dose in combination with the other recommended prophylactic antiemetics, and reserve an additional 4 mg i.v. dexamethasone for rescue if needed (i.e., refractory to other rescue mechanisms). Dexamethasone, or other steroids, should not be used for routine antiemetic prophylaxis in patients with diabetes mellitus, but long-term adverse events, such as adrenal suppression and delayed/impaired wound healing, have not been seen with single bolus doses[67]. A recent meta-analysis by Leslie and Gan reviewed adverse events (AE) of monotherapy and combination therapy for PONV, and found that pooled odds ratios for AE were not significant for 5-$HT_3$/dexamethasone vs. dexamethasone except for headache ($OR_{pooled}$ = 1.75), and AE such as those noted above for dexamethasone (e.g., impaired wound healing) were not seen with combination therapy[73].

### Serotonin (5-$HT_3$) Receptor Antagonists

5-$HT_3$ receptor antagonists block the serotonin receptor in the chemoreceptor trigger zone. Ondansetron, granisetron, and dolasetron are the available 5-$HT_3$ antagonists in the U.S. Early studies with these drugs were conducted in patients on chemotherapy agents with increased emetogenic potential. These drugs lack the sedative and dysphoric effects of droperidol, the cardiovascular effects of the PTZ, and the EPS associated with high-dose metoclopramide. These attributes make them particularly favorable in outpatient surgery. Minor side effects include headache, light headedness, hypotension, and elevations in liver enzymes (ondansetron, dolasetron)[1]. A limitation to their use is their significant expense compared to traditional agents, although products are now becoming available in a generic form (e.g., ondansetron). The very low cost of generic ondansetron, along with the low cost of the aforementioned, multimodal generic medications, such as dexamethasone, and perphenazine or cyclizine, render routine multimodal antiemetics prophylaxis as a low-risk, low-cost, high-benefit opportunity**.** While dexamethasone has gained favor as a first-line antiemetic in moderate-risk patients due to its safety profile, efficacy, and low cost, we recommend ondansetron as a central part of multimodal prophylaxis with two or three agents, based on previous studies[11]. 5-$HT_3$ receptor antagonists are most effective when given at or near the end of the surgical procedure[70,74,75], even though product labeling indicates administration at the induction of anesthesia. Differences in efficacy among 5-$HT_3$ receptor antagonists have not been shown in clinical trials, nor have differences in side effect profile between the available 5-$HT_3$ agents designated one as superior over the other[1,11,48,70].

It should be noted that ondansetron and dolasetron are metabolized by CYP 2D6 (an isoform that may include a phenotype characterized by ultrarapid metabolism), whereas granisetron is metabolized by CYP 3A4[76]. In our opinion, if ondansetron has been preceded by a CYP 2D6 inhibitor (such as perphenazine and/or cyclizine), then a repeat 5-$HT_3$ antagonist dose, even with granisetron, may also likely be ineffective, but this concept requires further study. However, if a failure of PONV prophylaxis occurs with ondansetron or dolasetron in the absence of a CYP 2D6 inhibitor, then single-dose rescue with granisetron (0.1–1.0 mg i.v.) is appropriate. The less-likely drug interaction profile with granisetron in the elderly (related to the differing cytochrome P450 metabolism) may make granisetron a rescue 5-$HT_3$ receptor antagonist of first choice in the elderly. Again, the clinical significance of this pharmacodynamic interaction requires further investigation, but has been subject to study in published reviews[77].

### Aprepitant

Aprepitant is the only FDA-approved medication with extended duration (up to 48 h) for the prevention of PONV. Aprepitant is unique in that it selectively binds and antagonizes the substance P/Neurokinin-1 (NK-1) receptor[78]. The NK-1 receptor is highly concentrated in the vomiting center located in the brainstem, and antagonism of this receptor site leads to its antiemetic effect[78,79]. The NK-1 receptor has little or no affinity for the 5-$HT_3$, dopamine, histaminic, and corticosteroid receptors located in the CTZ. Because of aprepitant's different mechanism of action, it can be used by itself solely for prevention of PONV or in conjunction as multimodal therapy for prevention of PONV in patients at moderate to high risk for PONV.

Side effects are similar to ondansetron, and no serious adverse drug-related experiences were reported in PONV studies in patients taking aprepitant[80]. Currently, aprepitant is only available as an oral, nonparticulate preparation (40-mg capsule) and should be given within 1–3 h before induction of anesthesia for the prevention of acute and delayed PONV. The current cost of aprepitant seems likely to limit its routine use in patients with a low risk for PONV.

## ALTERNATIVE THERAPIES, SPECIAL CONSIDERATIONS, AND PATIENT POPULATIONS

### Acupuncture and Acupressure

In facilities with capabilities and expertise in acupoint stimulation, failure of recommended prophylaxis followed by immediate acupuncture consultation can be done. The most common type of acupoint stimulation is of the *P-6* acupoint in the hand. The proposed mechanism for this may involve endorphin release or serotonin changes. A 1999 meta-analysis of nonpharmacologic approaches for prevention of PONV showed that nonpharmacologic approaches were equally effective as commonly used antiemetics in preventing early and late PONV (NNT = 5)[81]. Two studies published in 2002 showed that acustimulation was comparable to ondansetron in prophylactic effect, and combining the pharmacologic and nonpharmacologic approaches was better than either agent separately[82,83]. Gan et al., in their PONV consensus guidelines, denote that nonpharmacologic therapies should be considered in patients at moderate-to-high risk for PONV[11]. We advocate repeated small doses (see Table 1) if the licensed acupuncturist will not be available within 15 min of the consultation, but if repeat rescue doses are successful, then acupuncture emphasis will be placed on preventing PDNV (e.g., recurrence during car travel and/or on arrival home).

Acupuncture and other stimulation of the *P-6* acupuncture point, located on the volar aspect of the wrist, has been associated with significant reduction of PONV. In their Cochrane Review and a previous meta-analysis, Lee and Done review studies of needle and other stimulation at *P-6*, which is commonly used in the treatment of nausea[81,84]. Similar impressions are derived from a more recent meta-analysis, which also showed that the effect size is comparable in magnitude to premedication with antiemetic medication[85]. Benefit was seen for postoperative nausea as well as vomiting, and was seen in children as well as adults. The improvement was seen regardless of whether the study investigated needle or nonacupuncture stimulation. Acupuncture was typically provided in the preoperative or immediate postoperative period, and studies looked at manual stimulation of the needles, i.e. twirling, as well as electrical stimulation. Nonacupuncture stimulation included acupressure with a wrist band containing a magnetic marble, an electric wrist band that provides transcutaneous stimulation, low-energy laser stimulation, and irritant injection with 0.2 cc of 50% dextrose. Auricular acupuncture and stimulation of Korean Hand Acupuncture points have also shown efficacy in the prevention of PONV[86,87].

From a practical standpoint, in most states, licensure is needed for physicians to practice acupuncture — typically requiring 200 or more continuing education hours. If a medical acupuncturist is part of a perioperative team, needles can be placed at *P-6*, either preoperatively or in the PACU. Given the impression that *de qi* or a needling sensation is necessary for the therapeutic benefit, it is preferable for the patient to be awake and responsive during the needle placement. Typically, the needles are left in place for 10–15 min and stimulated either by periodic manual stimulation or by connection to an electroacupuncture unit. The patient can be treated again on postoperative rounds.

Another option is for an acupuncturist to consult on the inpatient service, seeing the patient later in the first postoperative day, with subsequent treatment if needed. Typically, the session will last 20–30 min, and one may treat pain as well as nausea and vomiting. A protocol used in our facility is to first place needles at *P-6* with manual stimulation; then treat *LI-4* and *ST-36—2* (common analgesic points located on the hand and knee) with electrical stimulation, and finally treat the auricular point *Shen Men*. Additionally, one may include one or two points targeting Chinese meridians pertinent to the organs treated in the surgery. However, caution is advised not to treat too many points or stimulate the needles aggressively, especially in frail or older patients. While this reflects practice at our institution, and allows us to treat nausea/vomiting, pain and postoperative stress, this more comprehensive approach has not been studied systematically.

Although the needling is usually not painful, given that children may already be anxious about the surgery experience, the noninvasive approaches described above may be particularly helpful in a pediatric setting. Additionally, these approaches can be used if there is no medical acupuncturist immediately available at the time of surgery. Options include acupressure with wrist bands or transcutaneous electrical stimulation. These bands should be placed preoperatively and left in place for 12–24 h.

### Hyperemesis Gravidarum

Hyperemesis gravidarum (HEG) is a rare disorder in pregnancy that is a severe variant of the more common “morning sickness” that usually occurs in the first trimester of gestation[88]. Understanding the dilemma posed by HEG may provide future research insights for the treatment of PONV in outpatients both in-hospital and at home. Since it occurs early in pregnancy, there is usually a reluctance to prescribe antiemetic medication due to the concern for developmental effects on the fetus. Organogenesis occurs up to the 8th week of gestation, and administration of any medication, including antiemetics, can have an effect on fetal organ development. The only antiemetic that is classified as Category A during pregnancy (controlled studies in women fail to demonstrate a risk to the fetus in the first trimester and later trimesters) is doxylamine, the antihistamine that is the active ingredient in some over-the-counter cold preparations[89]. Treatment of HEG is primarily supportive, and can include active hydration and vitamin supplementation, even home hyperalimentation. Common antiemetics used currently are the 5-$HT_3$ antagonists, which are all classified as Category B (no controlled studies have shown demonstrated risk in pregnancy) along with other Category B antihistamines, such as cyclizine and dimenhydrinate. In early pregnancy, perphenazine, droperidol, and haloperidol have been used for HEG, and are assigned to Category C (animal studies have demonstrated risk, *or* no controlled studies are available; to be used only if potential benefits justify potential fetal risks). Dexamethasone, pulsed steroids[90], and scopolamine have also been used for HEG, and all are assigned to Category C. Maternal dystonic-like reactions can occur after the use of benzamides, butyrophenones, PTZ, *and* possibly 5-$HT_3$ receptor antagonists. Recently, a report of dystonic reaction created by prochlorperazine, metoclopramide, and/or ondansetron was unmasked by the removal of a scopolamine transdermal patch[91].

### C-Section/Parturients

Recent findings in cesarean section (C-section) patients may be helpful in future studies of outpatients. Parturients are not outpatients, but like outpatients, they commonly wish to maintain or regain (as soon as possible) alertness, to facilitate bonding with their newborns. Much of the PONV encountered in the C-section population relates to blood pressure issues, peritoneal tugging, and/or narcotic overdosing. Most pharmacologic studies addressing PONV in C-section patients involve administration of the study medication after clamping of the umbilical cord.

Prophylactic ephedrine reduces hypotension induced by spinal anesthesia, but has no effect on PONV[92,93]; however, glycopyrrolate (0.2 mg) reduces both ephedrine requirements and PONV[94]. Patients receiving volume loading with hydroxyethyl starch (500 ml) have less hypotension and less PONV than those receiving lactated Ringer’s solution (1 l)[95]. Propofol (1 mg/kg/h) appears to be an optimal dose for PONV prevention[96], and is more effective than placebo[97], equiefficacious with droperidol (1.25 mg)[98], and propofol’s PONV effects are augmented by dexamethasone (8 mg)[99]. Droperidol is more useful in preventing intraoperative nausea and vomiting, but not PONV[100]. Ondansetron is more efficacious than placebo or metoclopramide[101]. Specifically, ondansetron appears to reduce the severity (but not the incidence) of nausea, and conversely appears to reduce the incidence (but not the severity) of vomiting[102]. Granisetron (3 mg) prevents both intraoperative and postoperative nausea and vomiting[100], is more effective as monotherapy than placebo or droperidol[103], and the antiemetic effect of granisetron is enhanced by dexamethasone (8 mg)[104]. The efficacy of high-dose granisetron (but lower efficacy of conventionally dosed ondansetron) appears to be likely related to greater increase of CYP 2D6 (by 50%) vs. CYP 3A4 (by 35%) during the last 4 weeks of pregnancy[105]. Cyclizine is more efficacious than dexamethasone for intrathecal morphine-induced PONV[54], while ondansetron (8 mg) is better than placebo for intrathecal opioid-induced PONV[106]. The scopolamine patch is effective in reducing PONV after C-section under epidural anesthesia (including preservative-free morphine in the epidural injection)[107]. Results regarding acupuncture and acupressure techniques appear to be inconclusive at this time[108,109]. There may be promise for intrathecal midazolam reducing both postoperative pain and PONV[110].

From this review, it appears that future study adopting a “zero-tolerance” approach in parturients can be directed toward the use of single-dose oral cyclizine and/or perphenazine (which are labeled as category B and C, respectively, for use in first-trimester HEG), intravenous glycopyrrolate and hydroxyethyl starch, followed by low-dose propofol (1 mg/kg/h), granisetron, and dexamethasone (5–8 mg). The cyclizine-or-perphenazine oral premedication (CYP 2D6 inhibitors) would be unlikely to enhance the efficacy of granisetron (a CYP 3A4 substrate), but the use of CYP 2D6 inhibitors may render ondansetron or dolasetron (both CYP 2D6 substrates) more efficacious. The principle hypothesis for future study would be to determine if this multimodal combination can prevent PONV related to all three generally accepted PONV mechanisms in C-section patients (opioid-related, spinal-induced hypotension in the setting of a gravid uterus and partial compression of the inferior vena cava, and peritoneal traction).

### SUMMARY AND ALGORITHM FOR CARE

There continues to be a wealth of information published about PONV prevention and treatment, available treatments, risk assessment, and algorithms for care. Despite this, patients continue to complain and experience PONV. It has been shown that patients have a minimum PONV risk of 10%, and in ambulatory surgery, risk factor assessment denotes nearly all patients having at least one, if not two, independent factors for PONV. Our experience with routine multimodal prophylaxis in ambulatory surgery is the basis for our recommendations — a “zero-tolerance” antiemetic algorithm, involving routine prophylaxis by first avoiding volatile agents and opioids to the extent possible, using locoregional anesthesia and multimodal analgesia, and low doses of three nonsedating traditional antiemetics with a fourth agent added if additional risk factors are present. We do not advocate repeat dosing of any particular antiemetic mechanism, except for possibly prescribing granisetron after other 5-$HT_3$ antagonists were used (in the absence of pretreatment with a CYP 2D6 inhibitor, such as perphenazine, cyclizine, and/or haloperidol), due to potential cytochrome P450 interactions. Fig. 2 describes our suggested approach for PONV prophylaxis and rescue treatment in the ambulatory surgery setting, based on a “zero-tolerance” objective while reviewing the literature.
